# An Overview of the Recent Progress in Modifications of Carbon Nanotubes for Hydrogen Adsorption

**DOI:** 10.3390/nano10020255

**Published:** 2020-02-01

**Authors:** Jinzhe Lyu, Viktor Kudiiarov, Andrey Lider

**Affiliations:** Division for Experimental Physics, School of Nuclear Science & Engineering, National Research Tomsk Polytechnic University, Lenin Ave. 43, Tomsk 634034, Russia; victor31479@mail.ru (V.K.); lider@tpu.ru (A.L.)

**Keywords:** hydrogen, modification of CNTs, physisorption, chemisorption, activation, spillover mechanism, Kubas interaction

## Abstract

Many researchers have carried out experimental research and theoretical analysis on hydrogen storage in carbon nanotubes (CNTs), but the results are very inconsistent. The present paper reviewed recent progress in improving the hydrogen storage properties of CNTs by various modifications and analyzed the hydrogen storage mechanism of CNTs. It is certain that the hydrogen storage in CNTs is the result of the combined action of physisorption and chemisorption. However, H_2_ adsorption on metal-functionalized CNTs still lacks a consistent theory. In the future, the research of CNTs for hydrogen adsorption should be developed in the following three directions: (1) A detailed study of the optimum number of metal atoms without aggregation on CNT should be performed, at the same time suitable preparation methods for realizing controllable doping site and doped configurations should be devised; (2) The material synthesis, purification, and activation methods have to be optimized; (3) Active sites, molecular configurations, effectively accessible surface area, pore size, surface topology, chemical composition of the surface, applied pressure and temperature, defects and dopant, which are some of the important factors that strongly affect the hydrogen adsorption in CNTs, should be better understood.

## 1. Introduction 

At present, the problems of environmental protection and energy storage are increasingly becoming the focus of attention [1,2,3]. At the same time, countries all over the world are making efforts to develop new clean energy, among which hydrogen, as an efficient energy carrier, has received considerable attention due to its abundant resources and significantly reduced impact on the environment [4,5,6]. Hydrogen has some characteristics that other energy sources do not have: (1) Hydrogen is renewable. Hydrogen generates electricity or heat through chemical reactions and is accompanied by the production of water, which can be converted into hydrogen and oxygen by electrolysis; (2) Hydrogen is storable. Hydrogen can be stored on a large scale as easily as natural gas. This is the biggest difference between hydrogen energy and electric energy or thermal energy. Electricity or heat can be stored in the form of hydrogen in places and periods with excess electricity. This also makes hydrogen play the role that other energy carriers cannot play in the application of renewable energy; (3) Hydrogen energy is one of the most environmentally friendly energy sources. In cryogenic fuel cells, hydrogen is converted into electricity and water by electrochemical reaction without emitting ${\mathrm{CO}}_{2}$ and ${\mathrm{NO}}_{x}$. In addition, the use of the hydrogen-fueled internal combustion engine is also an effective way to significantly reduce pollution [7,8,9,10]. The research and development of hydrogen energy mainly include three main parts: hydrogen production, hydrogen storage, and hydrogen use. With the continuous breakthrough of the practical technology of proton exchange membrane fuel cell (PEMFC), the problem of the storage and transportation of hydrogen, especially the problem of an on-board hydrogen source, has become the bottleneck of fuel cell technology in practical application. The target proposed by the U.S. Department of Energy (DOE) for an on-board hydrogen storage system is that the weight hydrogen storage density should be no less than 6.5 wt%, and the volumetric hydrogen storage density should be no less than 63 kg/m^3^ in the range of pressures up to 100 bar [11,12]. Traditional hydrogen storage technologies, such as high-pressure hydrogen storage and liquid hydrogen, cannot meet these two conditions at the same time, so researchers focus on other new hydrogen storage materials. At present, the known on-board storage and transportation methods are usually divided into three categories: solid, liquid, and gaseous, of which both liquid and gaseous state hydrogen storage refer to the form of hydrogen itself, while solid-state hydrogen storage refers to the shape of the hydrogen storage medium. The main studied solid hydrogen storage materials are carbon nanotube (CNT), metal hydride, borohydride, aluminum hydride, and so on [13,14,15].

In 1997, Dillon et al. [16] first reported the high hydrogen storage capacity of single-walled carbon nanotubes (SWCNTs), achieving 5 wt%–10 wt% under the condition of 273 K and 0.04 MPa, which could meet the hydrogen storage target set by US DOE, thereby they considered CNTs to be an ideal hydrogen storage material. Many researchers have carried out experimental research and theoretical analysis on hydrogen storage in CNTs, but the results are very inconsistent [12,17,18,19,20,21,22,23,24,25,26,27,28,29,30,31,32,33,34,35,36,37], some have shown that CNTs meet or exceed target set by US DOE [31,32,33,34], while others reject CNTs or even considered CNTs not suitable for hydrogen storage [35,36,37]. The main reason for the great difference in the research results of hydrogen storage in CNTs is the influence of internal and external factors on the hydrogen storage properties of CNTs. Internal factors refer to the characteristics of CNTs, such as specific surface area, opening degree, purity, etc. [38], while external factors refer to measurement methods, testing devices, test gases, ambient temperature, pressure stability, etc. [12,35,39,40,41]. Because the mass of the samples used in most experiments is small, the influence of the above-mentioned factors on the measured hydrogen storage properties of CNTs is more significant, which leads to many disputes about the hydrogen storage properties and mechanism of CNTs in previous studies. The controversy over the hydrogen storage properties is related to whether CNTs are good hydrogen storage materials [42], while the most important controversy over the mechanism of hydrogen storage is whether the nature of hydrogen storage behavior of CNTs is chemisorption or physisorption, or the coexistence of the two kinds of adsorption [43,44,45,46,47]. In fact, these two kinds of controversies are not independent of each other, and the relationship between them lies in the fact that [13]: (1) when the hydrogen storage capacity is small, the hydrogen desorption temperature is low, or the calculated adsorption heat is low, researchers often infer that only physisorption occurs in the adsorption process, but when partial adsorbed hydrogen is released at high temperature, or the hydrogen storage capacity calculated by physisorption model is less than the experimental value, researchers believe that chemisorption also occurred in the adsorption process; (2) Some researchers believe that there is only physisorption in CNTs due to not finding new chemical phase after hydrogen adsorption of CNTs [43], while some researchers have found new chemical phase [45]. From this, a new chemical phase and the part of hydrogen released at a high temperature are considered to be the consequence of chemisorption of hydrogen [45,46]. 

Grand canonical Monte Carlo (GCMC) method is usually used to simulate physisorption [13,48,49,50], while the density functional theory (DFT) is usually used in chemisorption simulation [51,52,53,54]. The hydrogen storage capacity of CNTs predicted by different models is different, so it is necessary to study the adsorption mechanism of CNTs. At present, it is widely believed that similar to $A_{m}B_{n}$ hydrogen storage alloys, the hydrogen adsorption in CNTs is the sum of irreversible chemisorption with high adsorption heat and reversible physisorption with low adsorption heat, which determines the fast kinetics and reversibility of hydrogen adsorption in CNTs [12,33,50,55,56,57,58,59,60,61,62,63,64,65]. The strongest proof of chemisorption in carbon nanotubes is the requirement of high temperature to completely remove hydrogen from hydrogenated CNTs [55]. Although it has been reported that the hysteresis phenomenon between the adsorption/desorption isotherms of CNTs can also suggest the chemisorption [12], Barghi et al. [66] attributed hysteresis to the metal impurities formed during CNT synthesis and/or the multilayer structure of the CNTs. 

Although the hydrogen storage properties of CNTs have been reported to be very different, there is an extremely great demand for hydrogen energy applications in electric vehicles, batteries, and military fields. Furthermore, CNTs play an important role in energy storage devices, such as photovoltaic cells, supercapacitors, and lithium-ion batteries [67,68,69,70,71,72,73,74,75,76]. Thus, investigations of CNTs as candidate hydrogen storage materials will not stop in the next few decades. The result of work with pristine unmodified CNTs has been somewhat disappointing, and researchers have turned their attention to modified CNTs. This has led to a variety of investigations dealing with modification of carbon materials like physical treatment, chemical treatment, loading various dopants (metals, metal oxides, hydrides, and hetero-atoms), CNTs in various geometrical forms, and phase purity. Based on the above aspects, this paper overviewed the research results of modifications of CNTs for hydrogen adsorption in recent years. Some factors affecting the hydrogen adsorption properties of CNTs were analyzed emphatically, and the key directions of CNT research in the future were pointed out.

## 2. Factors Affecting the Hydrogen Adsorption Properties of CNTs

### 2.1. Effect of Internal Factors on Hydrogen Adsorption Capacity of CNTs

**Figure 1:**
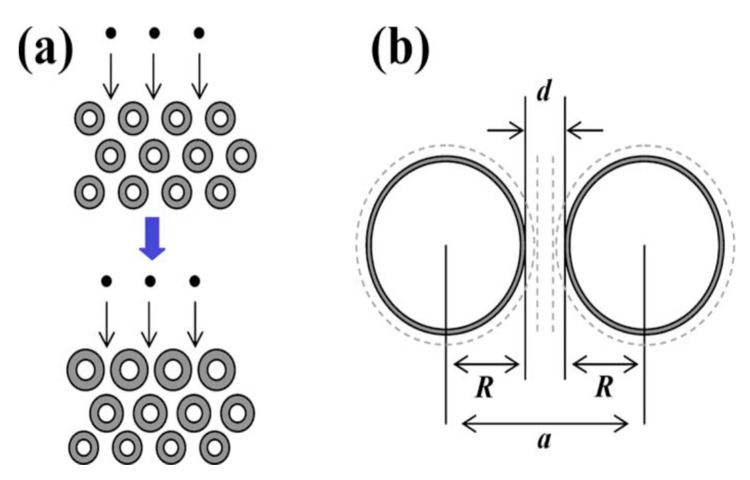
(**a**) Swelling of single-walled carbon nanotube (SWCNT) bundles during hydrogenation. (**b**) Characteristic dimensions of the bundle, namely, SWCNT radius, R, SWCNT center-to-center distance, a, and the intertube spacing, d. The dashed lines are used to indicate deformation due to SWCNT swelling upon hydrogenation [89].

The surface of the adsorbent is the area where adsorption occurs, and the most important properties for adsorption are the distribution characteristics of surface adsorption potential energy and the size of specific surface area. The adsorption potential of the solid surface determines the magnitude of the adsorption force, thereby affecting the adsorption capacity, and considering the surface topology of CNTs, the adsorbed molecules are always preferentially adsorbed on some specific sites [59,77] with the deepest potential well on the surface. Three different areas of bonding have been proposed where the H_2_ molecular axis is positioned: (i) parallel and above C–C bond, (ii) perpendicular and above carbon ring, and (iii) parallel and above carbon ring, respectively [3,33]. Although the mechanism of hydrogen adsorption in CNTs is not very clear, it is commonly agreed that hydrogen can be stored in the interior surfaces of CNTs, or on the exterior surface, or between the nanotubes in the case of CNT bundles [60,78]. After the adsorbent is given, the factors that determine the surface potential energy are the types and distribution of surface functional groups [79,80] and the size of surface curvature. When the type and distribution of the surface functional groups are determined, it is obvious that small surface curvature of the CNT leads to strong superposition potential produced by the adsorption potential of each independent functional group, thereby enhancing the adsorption of hydrogen molecules. The curvature is one of the reasons for the influence of tube diameter on the radial distribution of hydrogen in CNTs. When the tube diameter is large, the tube curvature is small, and the number of carbon atoms acting on hydrogen in the unit solid angle along the radial direction is little, which weakens the effect of carbon on hydrogen [81]. The specific surface area has a greater effect on the adsorption—the maximum gravimetric excess adsorption density varies linearly with the specific surface of the adsorbent [82]. Since the surface area of the solid adsorbent is composed of the outer surface and the inner surface of the pores, the specific surface area of the adsorbent depends on the size and distribution of the pore [83]. With this in mind, one can improve the hydrogen adsorption capacity of CNTs by experimentally controlling the geometric characteristics of CNTs, such as tube diameter, tube arrangement, the intertube spacing of SWCNT bundles, the wall-to-wall distance of multi-walled carbon nanotube (MWCNT), wall number of MWCNT, etc. Many investigations have provided theoretical and experimental evidence for this possibility [81,84,85,86,87,88]. Ghosh et al. [84] performed molecular dynamics (MD) simulations in the attempt to compare the hydrogen adsorption capacity of SWCNTs and SWCNT bundles with the intertube spacing less than 0.1 nm at 80 K and 298 K, respectively. The results have shown that isolated SWCNTs demonstrate higher hydrogen adsorption capacity at a low temperature, while SWCNT bundles have higher hydrogen adsorption capacity at room temperature, even higher than that of SWCNTs at 80 K, which suggests the significant advantage of SWCNT bundles over isolated nanotubes in terms of on-board hydrogen storage. The low hydrogen adsorption capacity of SWCNT bundles at low temperatures can be explained considering that intertube spacing (d) is smaller than the adsorbed layer thickness of hydrogen at a low temperature, causing poor space effect, thereby hydrogen cannot be captured in the interstitial spaces within the bundle. The fact that SWCNT with small intertube spacing is not suitable for the storage of hydrogen is in agreement with the results presented by Minami et al. [32]. Ghosh et al. [84] also reported that the adsorption capacity of the square array is considerably higher than that of the triangular array for all separation distances, which was attributed by the authors to the loose packing of nanotubes that results in larger cavities where hydrogen can be captured. Furthermore, it was pointed out by the authors that the hydrogen adsorption capacity of SWCNT bundles increases with the increase of the intertube spacing. This result is in accordance with the results reported by other groups [87]. The investigation of the swelling phenomenon (Figure 1) of SWCNT bundles during hydrogenation performed by Muniz [89] reveals that nanotube swelling can hinder the diffusion of hydrogen in the intertube spacing of SWCNT bundles during hydrogenation, thus reducing the adsorption of hydrogen in CNTs. This hindrance is more obvious in the case of high-density carbon nanotube bundles, which reflects the effect of the intertube spacing on the hydrogen adsorption capacity of SWCNT bundles, as reported by [84,87].

The relationship of the tube diameter and the radial distribution of hydrogen in SWCNT was investigated by Yan et al. [81] through GCMC calculations. It was found that for the SWCNT with small tube diameter, circular distribution of hydrogen molecules in the tube is observed, and the closer to the tube wall, more hydrogen molecules are adsorbed. When the tube diameter is large, the circular distribution of hydrogen near the tube wall is obvious, but the distribution of hydrogen far from the tube wall is chaotic. Different from the radial distribution, hydrogen is uniformly distributed along the tube axis. The same conclusion was made by Zhang et al. [88] through GCMC and MD. The distribution characteristics of hydrogen in CNTs were considered to be related to the potential well effect of carbon atoms on hydrogen molecules and the interaction between hydrogen molecules: (1) In the case of large tube diameter, the hydrogen near the tube wall is located in the deep area of "carbon potential well" and "hydrogen potential well", exerting strong binding to hydrogen, so the circular distribution is more obvious, which constitutes the potential effect in CNTs; (2) The hydrogen located far away from the surface area of CNTs lies in the shallow areas of "carbon potential well" and "hydrogen potential well", it is difficult for which to bind hydrogen stably; therefore, the distribution of hydrogen is relatively messy, which constitutes the space effect in CNTs [87,90,91]. In addition, Zhang et al. [88] performed GCMC and MD on various CNTs to study the effects of the arrangement and the intertube spacing of CNTs on the radial distribution of hydrogen and the hydrogen adsorption capacity of CNTs. The simulation results of hydrogen adsorption capacity of CNTs suggested that: (1) For a given tube diameter, the SWCNT has the same hydrogen adsorption capacity as the SWCNT in the bundle (that is, the hydrogen adsorption capacity of SWCNT has nothing to do with the arrangement of the bundle), which is in agreement with the results presented by [87]; (2) When the intertube spacing is greater than a certain value, the hydrogen adsorption capacity of the intertube spacing is greater than that of the internal part of the tube, which is also confirmed by [87]; (3) Compared with the SWCNT bundle arranged in the square array, the triangular array SWCNT bundle with the same tube diameter and the intertube spacing has greater hydrogen adsorption capacity, which is in contrast to the findings of [87] and [84]. This difference was explained by Minami et al. [32] through GCMC simulation. The simulation results revealed that structural optimization of arranged SWNT for hydrogen adsorption is associated with inter-axis distances and pressure; when the pressure is greater than 1 MPa, the hydrogen adsorption capacity of SWNTs with a square lattice structure is greater than that of SWNTs with a triangular lattice structure; when the pressure is less than 1 MPa, the opposite is true; (4) For MWCNT, for a given wall-to-wall spacing, the hydrogen adsorption capacity decreases with the increase of the number of layers, and for a given number of layers, the hydrogen adsorption capacity increases with the increase of wall-to-wall spacing; (5) The hydrogen adsorption capacity of SWCNT is larger than that of MWCNT with the same tube diameter. 

In the recent research work of Brzhezinskaya et al. [92] on changes in the structure of SWCNT bundles caused by hydrogenation, it was claimed that chemical covalent bonds between hydrogen atoms and SWNT walls arise with the formation of the $C_{4}H$ and $C_{2}H$ phases. However, not the entire surface of the SWNTs located inside the bundles is accessible for hydrogenation but only segments adjacent to triangular pores. The influence of tube diameter and chirality on the chemisorption of hydrogen in CNTs was investigated by Wang et al. [93] through the self-consistent-charge density-functional tight-binding method. The results have shown that hydrogenation energy is positively related to the inverse of the square of the tube diameter, and compared with the zigzag structure, the armchair structure leads to higher binding energy for the given diameter of CNTs. Dependence of chemisorption of hydrogen on tube diameter and chirality was also observed by Muniz [89] et al. Unlike chemisorption, the chirality of CNTs has little effect on their hydrogen storage capacity by physisorption [88]. 

The size effect of CNTs has also been found to affect the hydrogen adsorption. CNTs are categorized by Chen et al. [11] as tiny, small, medium, and large CNTs. It was found by the authors that tiny CNTs could not achieve the goals of hydrogen storage set by US DOE without fracture, small CNTs are strained during hydrogen adsorption, medium CNTs can achieve the above goals without the strain and do not self-collapse, and large CNTs may self-collapse upon the release of hydrogen. It should be noted that the geometric characteristics of CNTs have different effects on their weight hydrogen adsorption capacity and volume hydrogen adsorption capacity. For example, Minami et al. [32] reported that in the case of foxed inter-axis distances and at 77 K 1 MPa volume, hydrogen adsorption capacity of SWCNT bundles increases with the increase of tube diameters and the decrease of inter-axis distances, while the relationship between weight hydrogen adsorption capacity and tube diameters/inter-axis distances is not monotonous.

### 2.2. Effect of External Factors on Hydrogen Adsorption Capacity of CNTs

Temperature and pressure are the two most important parameters affecting the hydrogen adsorption capacity of CNTs [94,95,96,97]. The work of Zhao [95] is aimed at investigating the effects of temperature and pressure on hydrogen adsorption properties of SWCNTs through the MD simulation method. The investigation reveals that for the given tube diameter and temperature, the hydrogen adsorption capacity of SWCNTs increases with the increase of pressure, which is in agreement with the results presented by [96]; for the given tube diameter and pressure, the hydrogen adsorption capacity of SWCNTs decreases with the increase of temperature; for the given temperature and pressure, the hydrogen adsorption capacity of SWCNTs decreases with the decrease of tube diameter. Hydrogen molecules tend to form a ring (circular distribution) near the tube wall. At low temperatures, with the increase of pressure in SWCNTs with large tube diameter, not only the number of hydrogen molecules on the original ring increases but also the tendency to form smaller and smaller rings will be observed. Whereas in SWCNTs, with small diameter occurs only monolayer adsorption with the increase of pressure, i.e., another hydrogen ring will not be formed no matter how high the pressure is. At room temperature, regardless of the tube diameter, with the increase of pressure only, an increase of the number of hydrogen molecules on the original ring will be observed without the formation of another hydrogen ring. The hydrogen molecules in the middle of the ring are just disorderly arranged. The simulation results show that the increase of the hydrogen adsorption capacity of CNTs with the increase of pressure is determined by physical adsorption, which is in accordance with the recent experimental results reported by other groups [97,98]. Zhao [95] presented the mechanism of the effect of temperature and pressure on the hydrogen distribution in CNTs. It was pointed out by the author that because there are several axially symmetrical potential wells in the hydrogen-adsorbed CNTs, and the hydrogen molecules always gather to the low energy area, a clear hydrogen ring structure is formed at the potential well. The closer to the tube wall, the deeper the potential well is, the greater the numerical density of hydrogen molecules becomes. At low temperature, due to the small kinetic energy, it is not easy for hydrogen molecules to break free from the bondage of the potential well, so multi-layer hydrogen rings can be formed. At high temperatures, the hydrogen molecular kinetic energy is so high that hydrogen molecules can only form one hydrogen ring near the tube wall. Under low pressure, because the number of hydrogen molecules in the tube is less, the statistical level may not be accurate, the molecular arrangement observed is relatively scattered, while the amount of hydrogen molecules in the tube is enough under high pressure, so the statistical level is accurate, and the second hydrogen ring can be observed obviously. SAFA et al. [99] revealed the temperature dependence of physisorption and chemisorption by elastic recoil detection analysis: (1) H_2_ physisorption mainly occurs at cryogenic temperatures; (2) In the range of 30 ≤ T ≤ 100 °C, with increase of temperature, H_2_ desorption is the predominant phenomenon; (3) At temperature about 100 °C to 300 °C, hydrogen molecules gain enough energy to overcome activation energy barrier for breaking up H–H bond. Hydrogen atoms sharing their electron with CNT atoms are then chemisorbed by CNTs. Therefore, the increase in hydrogen adsorption capacity at this temperature range could be considered as the consequence of the chemisorption process. Moreover, some adsorption sites in CNTs with higher adsorption energies could become active at this temperature range. The decrease of hydrogen concentration at elevated temperatures is related to the desorption of chemical adsorbed hydrogen, which was also reported by Ströbel et al. [100]. In view of the phenomenon that at room temperature hydrogen molecules with high kinetic energy are easy to break free from the van der Waals force of CNTs, Liu et al. [101], using MD simulations, presented a simple way of preventing the physically adsorbed hydrogen molecules from escaping from CNTs at room temperature by bending CNTs so as to increase the hydrogen adsorption capacity. Their simulation results showed that bending the CNTs to the critical angle allows encapsulating a large number of hydrogen molecules, which are put in CNTs at low temperatures. The critical angle decreases with the increase of CNT length, while it increases with the increase of hydrogen number and temperature. Furthermore, bending CNTs as well leads to the change of the binding energy between hydrogen and CNTs by changing the curvature of CNTs, thus affecting the hydrogen adsorption capacity of CNTs [102]. Influences of other external factors on the hydrogen adsorption performance of CNTs have been reported in detail in work [13].

## 3. Modification of CNTs

### 3.1. Activation of CNTs

According to the characteristics of physisorption, the adsorption capacity of a physisorption-based hydrogen carrier is the sum of the capacity due to adsorption on a solid surface and the capacity due to compression in the void space [103]. It is noteworthy that the hydrogen molecules due to physisorption on the solid surface are arranged in a single layer on the carbon surface; a high surface area provides a great number of adsorption sites for hydrogen. Therefore, the hydrogen adsorption capacity of CNTs is proportional to the specific surface area [104]. The molecular-sized pores can absorb a large amount of hydrogen, so pore size and porosity exert a great influence on the hydrogen adsorption capacity of CNTs [105,106,107]. The defects on the surface of CNTs not only serve as the entry point of the hydrogen but also shorten the diffusion path as well as increase the surface area and pore volume of CNTs, thus increasing the hydrogen adsorption capacity. In addition, defect sites like nanometer-sized pores create deeper potential wells, which enhance the interaction between hydrogen and CNTs, thereby increasing the average adsorption binding energy (*E_ad_*) of H_2_ [84,107]. Activation is an effective method to increase the surface area and pore volume of CNTs and introduce defects as well as functional groups, which improve the hydrogen adsorption properties of the surface of CNTs. The commonly used activation methods of CNTs are divided into physical treatment [78,101,108,109,110,111,112,113,114] and chemical treatment [37,79,105,107,115,116,117,118]. 

#### 3.1.1. Physical Treatment

Ion irradiation is the frequently-used physical treatment method [108,109,119]. Silambarasan et al. [108] reported the effects of different γ irradiation doses on the structure and hydrogen adsorption capacity of MWCNTs. It was found out that the γ-irradiation-induced annealing reduced the structural imperfection of MWCNTs; at 100–150 kGy, with the increase of irradiation dose, direct interaction of Compton electrons with the atoms of the nanotubes led to increase in vacancy defects of MWCNTs, resulting in increase in hydrogen adsorption capacity to 1.2 wt.% at 100 °C and ambient pressure. High dose (200 kGy) made the structure of MWCNTs distorted. Geng et al. [109], eliminating the interference of moisture, found that microwave-treated MWCNTs adsorbed up to 0.35 wt% H_2_ over 0.1 wt% for the pristine sample and further up to 0.4 wt% after subsequent heat-treatment. The microwave-induced increase in the hydrogen adsorption capacity of CNTs was attributed by the authors to the introduction of micropore surfaces, while the improved hydrogen adoption capacity in the result of heat treatment was attributed by the authors to increase the crystallinity of MWCNTs, which might facilitate the hydrogen diffusion in the MWNTs. The dependences of hydrogen adoption capacity on micropore surfaces and crystallinity of MWCNTs are in agreement with the results presented by other groups [110,120]. The hydrogen adsorption properties of γ-irradiated SWCNT bundles in various gas media were investigated by Dolbin et al. [121]. The best hydrogen adsorption capacity was registered for γ-irradiated SWCNT bundles in hydrogen gas media, which was attributed by the authors to the high hydrogen chemisorption capacity researched under hydrogen atmosphere irradiation. 

Mechanical milling is another method to improve the hydrogen adsorption capacity of CNTs. Ball milling leads to the break of CNTs, shortening the length of CNTs and opening the ends of CNTs, as well as increasing the defects and the surface area, so as to increase the hydrogen adsorption capacity [78,111,112]. The investigation of Lee et al. [111] on effects of milling speed and milling time on hydrogen adsorption capacities of MWCNTs suggested that compared with milling time, milling speed exerts more influence on hydrogen adsorption capacities of MWCNTs. MWCNTs cryomilled at 700 rpm adsorbed more than approximately 22% H_2_ compared to that of unmilled MWCNTs due to the improvements in the specific surface area (17.4%) and pore volume (34.9%). Improvement of the effect of ball milling on hydrogen adsorption capacities of MWCNTs by using MgO was reported by Liu et al. [78]; it was found that the ball-milling time adopted to achieve almost the same hydrogen adsorption capacity of MWCNT could be reduced by 9 h by adding MgO as grinding aid.

#### 3.1.2. Chemical Treatment

**Figure 2:**
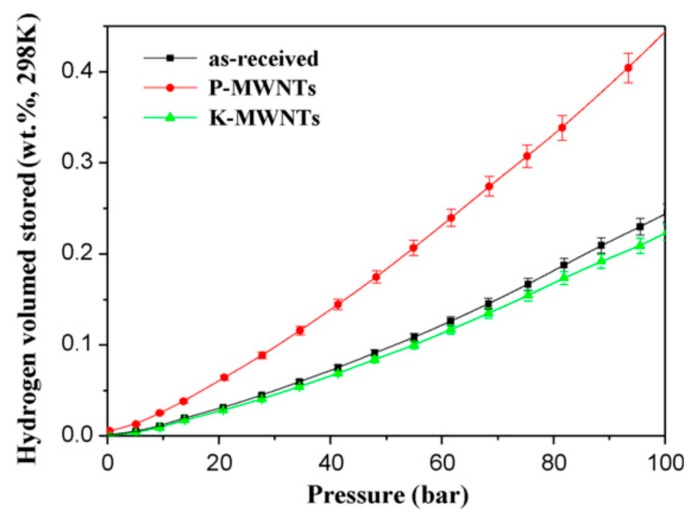
Hydrogen storage behaviors of the chemically-treated multi-walled carbon nanotube (MWCNTs), where P-MWCNTs are H_3_PO_4_-treated MWCNTs, K-MWCNTs are KOH-treated MWCNTs [79].

The commonly used chemical activation methods are acid treatment, basic treatment, and heat treatment. Lee et al. [79,80,105] systematically studied the effects of chemical treatments and activation temperature on hydrogen adsorption capacity of MWCNTs. As shown in Figure 2, when chemically-treated MWCNTs are not activated at a high temperature, only acidic treatment leads to the increase of hydrogen adsorption capacity, while basic treatment leads to the decrease of hydrogen adsorption capacity [79]. 

**Figure 3:**
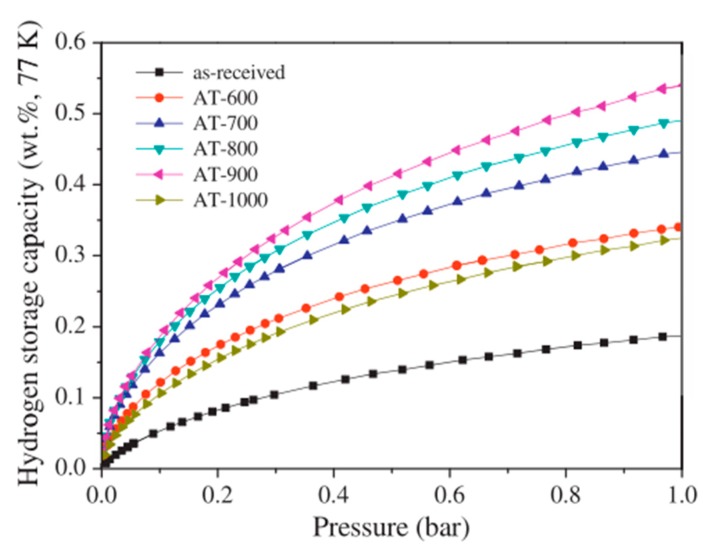
The hydrogen storage capacity of the KOH-activated MWCNTs at different activation temperatures (600 °C, 700 °C, 800 °C, 900 °C, 1000 °C), the weight ratio of KOH:MWCNT = 4:1 [105].

**Figure 4:**
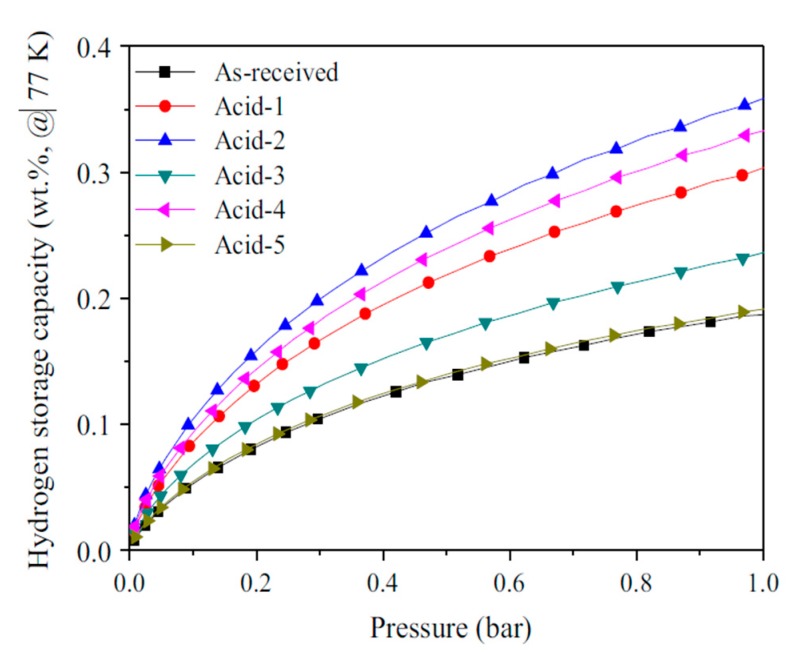
Hydrogen adsorption capacities of p-type MWCNTs with different acid-mixed treatments, where Acid-1 is H_2_SO_4_, Acid-2 is H_2_SO_4_:H_2_O_2_ = 3:1, Acid-3 is H_2_SO_4_:H_2_O_2_ = 1:1, Acid-4 is H_2_SO_4_:H_2_O_2_ = 1:3, Acid-5 is H_2_O_2_ [80].

It can be observed from Figure 3 that in the case of weight ratio of KOH:MWCNT = 4:1 0.54 wt%, hydrogen adsorption in MWCNTs activated at 900 °C was achieved at 77 K and 1 bar [105]. This value exceeds the maximum hydrogen adsorption capacity of acid-treated CNTs without heat treatment, as shown in Figure 4. The fact that heat treatment can enhance the effect of chemical treatment on the improvement of hydrogen adsorption capacity of MWCNTs was also reported by Elyassi et al. [118] by comparing hydrogen adsorption capacity of MWCNTs, which were activated by KOH at 900 °C and 3:1 weight ratio of $H_{2}{\mathrm{SO}}_{4}$:${\mathrm{HNO}}_{3}$ without heat treatment, respectively. In addition, Elyassi et al. [118] reported that KOH-activated MWCNTs with the weight ratio of KOH:MWCNT = 3:1 followed by 900 °C heat treatment exhibited 1.24 wt% hydrogen adsorption at 298 K and 34 bar, which was more than twice the hydrogen adsorption capacity reported in [105]. This suggests that the weight ratio of KOH:MWCNT has an effect on the hydrogen adsorption capacity of KOH-activated MWCNTs [107].

**Figure 5:**
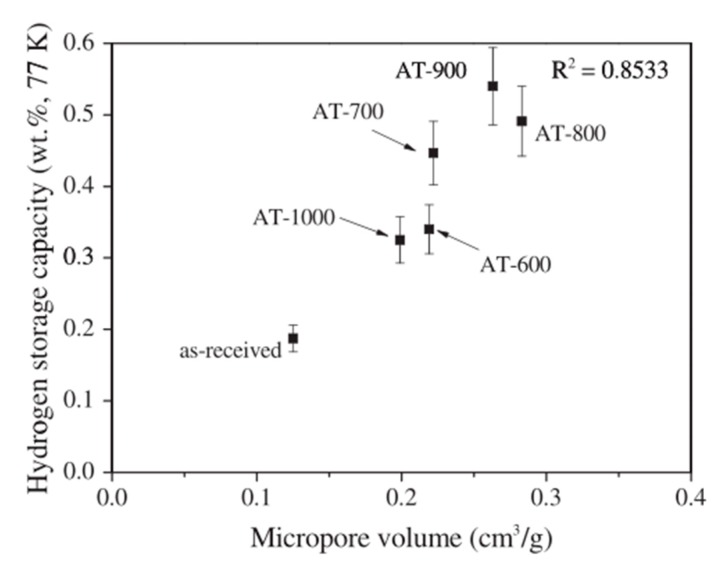
Relationship between the hydrogen adsorption capacity and micropore volume at different activation temperatures (600 °C, 700 °C, 800 °C, 900 °C, 1000 °C) [105].

Lee et al. [79,105] also found that with an increase of activation temperature, a large number of defects could be introduced in CNTs, and the specific surface area increases with the increase of activation temperature but decreases when the activation temperature increased to a certain value. Similar dependence on activation temperature was also observed in total pore volume, micropore volume (Figure 5), and hydrogen adsorption capacity. The authors attributed the dependence on the destruction of some of the micropores by excess chemical activation, leading to the formation of mesopores. The fact that with an increase of activation temperature, the hydrogen adsorption capacity and micropore volume of CNTs first increase and then decrease is in accordance with the results reported by Adeniran et al. [107], suggesting a close correlation between hydrogen adsorption capacity of CNTs and micropores [122,123].

In addition to acids and bases, bromine [115] and fluorine [116] are also used for the chemical treatment of CNTs. The increase in hydrogen adsorption capacity of bromine and fluorination-treated CNTs is not only due to the enlarged specific surface area and micropore volume as well as destroyed sp^2^ hybridization of CNTs, which increases hydrogen adsorption sites, but also due to attraction effects of induced bromine or fluorine groups on the electron in the hydrogen molecules as a result of the high electronegativity of bromine or fluorine compared with other acid/basic functional groups. 

Improvement in the hydrogen adsorption capacity can be achieved by increasing the purity of CNTs [106,124]; it was even reported that the adsorption capacity increases monotonously with the increase of purity of CNTs [3]. The effect of purity on the hydrogen storage of CNTs can be explained considering that metal particles impurity closes the opening of the CNTs, decreasing the exposed surface area of CNTs, thereby limiting the hydrogen adsorption [60,124,125].

Details of the investigation results of the part of the above selected works on activation of CNTs are presented in Table 1.

### 3.2. Loading Metals

**Figure 6:**
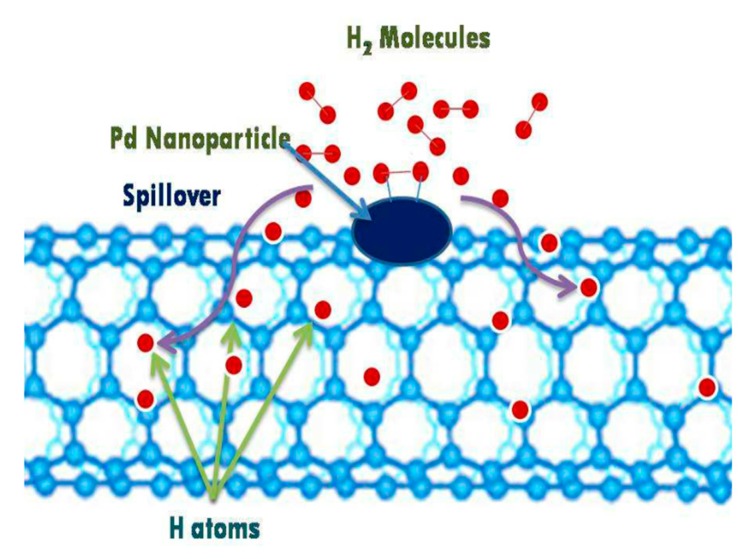
Schematic representation of hydrogen spillover mechanism on Pd dispersed carbon nanotube [130].

**Figure 7:**
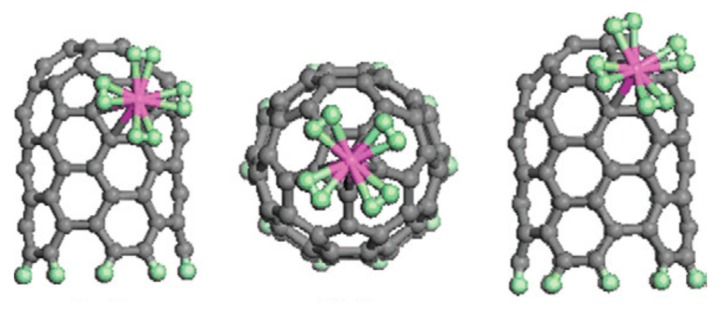
The Kubas type of interaction of adsorption of H_2_ molecules to a single Sc atom on different sites of SWCNT [134].

Theoretical and experimental results on hydrogen adsorption by CNTs with various metal dopants have been published. Although using the modified synthesis method without loading metals has been reported to facilitate the hydrogen adsorption capacity of CNTs [65,127], in fact, some residual metal catalyst has played an important role in the reported high capacities of hydrogen adsorption. Two possibilities explaining the catalysis effect of metal dopants on the hydrogen storage properties of CNTs have been reported: spillover mechanism (Figure 6) [122,128,129,130,131] and the Kubas type of interaction of hydrogenation (Figure 7) [47,132,133,134]. Through the spillover mechanism, the hydrogen adsorbed by the CNT-metal dopant system is more than the sum of hydrogen adsorbed by CNT and metal dopant separately under the same conditions. This can be explained considering synergistic effect [135,136,137,138] of constructed heterostructure of metal-CNTs in its hydrogen adsorption performance: (1) The number of adsorption sites for H is much greater than that for H_2_ [139]; (2) Part of the hydrogen atoms produced by the decomposition of hydrogen molecules on the metal catalyst will migrate to the carbon nanostructure surface. These H atoms that are now free from the H_2_ molecule can adhere to the edges of the tube wall, which contain unsaturated carbons, and then be chemisorbed into CNTs, thus increasing the hydrogen chemisorption capacity of CNTs without reducing or even promoting the physisorption of hydrogen molecules on CNTs [59,130,138,140,141,142]; (3) The strong interaction between metal catalyst and CNTs may facilitate hydrogen atoms spilling over from the metal nanoparticle surface to the defect sites on the nanotube, which strongly adsorb hydrogen atoms [60,140,142,143,144]. If the hydrogenation follows the Kubas-type interaction, the H_2_ molecules retain their molecular structure and do not dissociate to form metal dihydride [145,146,147]. Compared with hydrogen adsorbed by CNTs, *E_ad_* of H_2_ molecules adsorbed by metal-doped CNTs increases to 0.2–0.6 eV/$H_{2}$ caused by electron transfer from metal atoms to carbon atoms, which is between strong chemisorption and weak physisorption states and is ideal for the reversible adsorption/desorption of H_2_ at ambient conditions [47,102,148,149]. The increase in *E_ad_* is determined by metal dopants and the size effect of CNTs, as well as the interaction between hydrogen molecules, adsorbed on metal dopants [132,133,134]. It is worth emphasizing that 0.2 eV/H_2_ is the lowest requirement proposed by the U.S. DOE [150]. 

At present, the added metal catalysts are mainly Pd [117,125,129,130,135,140,141,143,151,152,153,154,155,156,157,158,159], Mg [160,161,162], Ni [98,136,139,163,164], Ti [34,137,138,165,166], Li [132,144,150,167], Al [102,168,169], etc.

Metal dopant content is one of the most important parameters to determine the catalytic effect on CNTs. The effect of metal dopant content on the hydrogen adsorption capacity of CNTs depends on the type of metal dopants. It was observed by Mehrabi et al. [163] that Ni-doped MWCNT prepared by chemical reduction exhibited the optimal hydrogen adsorption capacity when the Ni content was 13 wt%. The hydrogen adsorption experiments performed by Lee et al. [164] on CNTs doped with chemical reduction introduced nickels demonstrated that the hydrogen adsorption capacity increases at first and then decreases with the increase of Ni content. This data is different from the results reported by [130,151], where hydrogen adsorption capacity of Pd-doped MWCNT prepared by chemical reduction increases linearly with the increase of Pd content. It should be noted that the type of CNTs may also influence the dependence of hydrogen adsorption capacity on metal dopant content. Wu et al. [155] investigated the effect of Pd content (1 wt%, 2 wt%, 3 wt%) on the room temperature hydrogen adsorption capacity of double-walled carbon nanotubes (DWCNTs) prepared by chemical reduction. The best hydrogen adsorption capacity 3 wt% H_2_ and the maximum specific surface area 238.5 m^2^/g were registered for DWCNT–2 wt% Pd, which suggests that different from Pd-doped MWCNT, for DWCNT, the hydrogen adsorption capacity increases at first and then decreases with the increase of Pd content. The influence of doping methods on the effect of metal dopant content on hydrogen adsorption capacity of CNTs has been studied by several groups [151,153,155]. Studies of Wu et al. [153,155] showed that for DWCNT–2 wt% Pd, the hydrogen adsorption capacity of DWCNT–2 wt% Pd prepared by NaBH_4_ reduction was 3 wt%, while that of DWCNT–2 wt% Pd prepared by high temperature (500 °C) reduction was only 1.93 wt%. The investigation of Mehrabi et al. [151] revealed that the hydrogen adsorption capacity of Pd-doped MWCNT prepared by chemical reduction methods increases approximately linearly with the increase of Pd content, whereas the hydrogen content of Pd-doped MWCNT prepared by laser ablation shows the optimal value 1.1 wt% at 11.54 wt% Pd and decrease with further increase of Pd content. It was explained by the authors that long-time laser ablation causes great structural changes in MWCNT, while the structural changes caused by chemical reduction can be ignored. This kind of structural change was determined by Mortazavi et al. [154] as the merging of induced micropores, which form a larger area, leading to a drastic drop in the adsorption. It is reported [152] that Pd-doped MWCNT prepared by polyol methods exhibit better hydrogen adsorption properties than Pd-doped MWCNT prepared by wet impregnation. TEM studies of the samples reveal that polyol methods allow better dispersion of Pd particles on MWCNTs. Catalysis effect of Pd on CNTs can be improved by using additional stabilizing agents, which allow to reduce the size of nano-Pd particles, facilitate their uniform distribution on CNTs, and open the ports of CNTs, as well as enhance hydrogen spillover reaction [157,159], thus increasing hydrogen adsorption capacity.

It is widely admitted that the metal dopant content affects the hydrogen adsorption capacity of CNTs by exerting influence on the dispersion of metal dopants: (1) Proper content of metal dopants facilitate the spillover of hydrogen to CNT support, allowing the full contact between hydrogen molecules and the carbon structures, thereby significantly improving the hydrogen capacities of CNTs [130,139,155]; (2) Excess content of metal dopants leads to the aggregation of metal particles with the formation of large clusters, causing the blockage of the pores and effective surface area, thereby decreasing the hydrogen capacities of CNTs [134,141,147,155,163,170]. To achieve 100% hydrogenation via hydrogen spillover, it is also important that the metal catalyst is very evenly dispersed over the surface of the CNTs; this is only possible with nonbundled nanotubes, for which high metal-to-C dispersion can be achieved [171].

A single metal atom can bind multiple H_2_ molecules via the Kubas interaction, leading to high gravimetric and volumetric density, which is determined by the doping site and the doped configuration [137,150,165,172]. Liu et al. [150], using the unrestricted spin-polarized density functional theory, found that high H_2_ uptake of 13.45 wt% could be obtained by optimizing the number and position of Li dopants on CNT, i.e., eight Li dispersed at the hollow sites above the hexagonal carbon rings. SWCNTs with Stone–Wales defects and four stable Ti-doped configurations, in which the Ti atom is doped on the 5, 6, and 8 member rings of the Stone–Wales defect (1–3 configurations) and two carbon atoms of the 6-member ring are substituted by a Ti atom (4 configurations), were, respectively, simulated for their adsorption properties of hydrogen by Ghosh et al. [165]. MD simulation results showed that the fourth configuration allowed the optimum hydrogen adsorption properties—the gravimetric storage capacity of 7.75 wt% and the volumetric capacity of 209 g H_2_/L at room temperature and 650 atm. The theoretical investigations reveal that under the premise of suitable doping site and the doped configuration, the hydrogen adsorption capacity of metal-doped CNTs can be further improved by positively charging [167] or mechanical bending [34]. The improved hydrogen adsorption capacity of positively charged metal-doped CNTs can be explained by the increased binding energy of H_2_, which is attributed to the positively charged tube, improving the charge transfer from metal atom to the tube and making the metal atom more ionized, thereby strengthening the polarization interaction between H_2_ and metal atom [167]. Preparing CNTs in various geometrical forms is also considered to be the way to additionally increase the hydrogen adsorption capacity of CNTs. Tylianakis et al. [173] theoretically designed Super Diamond structured CNTs with the controlled location of the large pore, which could adsorb more than 20 wt% H_2_ at 77 K and adsorb up to 8 wt% H_2_ at room temperature. 

The doping effects of transition metals (Sc, Co, and Ni) on molecular adsorption of hydrogen in capped-SWCNTs were investigated by Tian et al. [134] through DFT calculations. Sc doping was found to yield the highest hydrogen adsorption capacity 7.08 wt% with the high binding energy per H_2_ of −0.93 eV. Investigating the hydrogen storage in MWCNTs decorated by Ca, Co, Fe, Ni, and Pd nanoparticles, Reyhani et al. [47] found that under ambient conditions, the best hydrogen adsorption capacity 7 wt% was registered for Pd-decorated MWCNT, whereas after six cycles of adsorption/desorption, the hydrogen adsorption capacity loss for the Pd-MWCNTs system was found about to be 55%, which was professed by the authors to the recrystallization of the defect sites on the carbon substrate [174]. It was noted that in spite of the relatively low hydrogen adsorption capacity (1.5 wt%) of Co-decorated MWCNT, after six cycles of adsorption/desorption, its capacity loss could be negligible. 

It has been observed by Seenithurai et al. [102] on the basis of DFT simulation that the decorating of Al atom allows an effective increase in *E_ad_* of SWCNTs. Single Al atom decorated on (8,0) SWCNT adsorbs up to six H_2_ molecules with an *E_ad_* of 0.201 eV/H_2_. Each Al atom in (8,0) CNT-8 Al adsorbs four H_2_ molecules, without clustering of Al atoms, and the adsorption capacity reaches to 6.15 wt%. No clustering of metal particles was attributed by Tian et al. [145] to the large binding energy between metal particles and CNTs, which acts as an energy barrier to prevent the clustering of metal particles. A layered structure of adsorbed H_2_ molecules on Al or AlH_3_-decorated SWCNT was found to realize high hydrogen adsorption capacity, which is much higher than the DOE’s target, whereas, in this case, *E_ad_* is small, only 0.1–0.2 eV/H_2_, which is not desirable for ambient condition applications [168,169]. 

Pt [122,123], Y [145,146], Ru [147] are also used as dopants in CNTs, and they all improved the hydrogen adsorption properties of CNTs to varying degrees.

Details of the investigation results of the part of the above selected works on modification by loading metals are presented in Table 2.

### 3.3. Loading Hetero-Atoms

The alternative of doping carbon materials with metals may be heteroatoms like N [175,176,177,178], P [179], Si [66,179], and B [19,180]. They seem to be promising as activators in heteroatom-containing carbon materials for hydrogen [181] adsorption application due to their properties like higher redox potential than that of carbon and the lower standard free energy of formation of hydrides, thereby reducing dissociation energy of hydrogen [176] or reducing the activation energy to chemisorption [179]. 

There are many factors that affect the activation effect of heteroatom doping, for example, gradation and the geometrical positions of hetero-atom doping. It was reported by Viswanathan et al. [176] that the dissociation energy of hydrogen for single boron substitution is 5.95 eV, whereas when two boron atoms are substituted in adjacent positions, the dissociation energy is reduced to 3.88 eV. It is further decreased to 0.28 eV when two boron atoms are substituted in the alternate positions. This implies that the substitution of boron at alternate positions is more favorable for hydrogen activation rather than substitution at adjacent positions. The types of carbon sources have also been reported to exert influence on the activation effect of heteroatom doping [19,176]. The recent hydrogen adsorption experiments performed by Ariharan et al. [177] demonstrated that CNTs doped with 8.5 wt% nitrogen could achieve 2 wt% hydrogen absorption at room temperature and 100 bar pressure. The GCMC calculation by Cho et al. [179] reported the unusual H_2_ adsorption capacity of Si-decorated SWCNTs compared with B, N, and P-decorated SWCNTs. When the doping amount of Si is 10 atom%, and the tube diameter is 4 nm, Si-decorated SWCNT has the maximum hydrogen adsorption capacity of 2.5 wt% at room temperature and 100 bar pressure. The dependence of the hydrogen adsorption properties of heteroatom-doped CNTs on the amount of heteroatom doping and the tube diameter was attributed by the authors to the dispersion, induced dipole, and quadrupole interactions.

Details of the investigation results of the part of the above selected works on modification by loading hetero-atoms are presented in Table 3.

### 3.4. Other Doping

Heteroatom-metal mixed doping has been theoretically reported to greatly improve the hydrogen storage properties of CNTs. Doping of B and N can increase *E_ad_* and the binding energy of metal atoms on CNTs without reducing the maximum number of hydrogen molecules that are chemically adsorbed on the Pt atom, thereby inhibiting metal atom from clustering on the surface of the CNT and improving the hydrogen storage capacity. However, it should be noted that too high H_2_ molecules’ average binding energy will make it difficult for reversible adsorption/desorption of hydrogen at ambient temperature and pressure [149,182,183]. Therefore, it is necessary to choose metal-doped CNTs with low *E_ad_* to carry out heteroatom doping modification, such as Li or Na [184].

In recent years, few works about hydrides [185,186,187,188,189] or metal oxide [190,191,192,193]-doped CNTs have been reported, and the catalytic mechanism of hydride or metal oxides on the hydrogen storage properties of CNTs is similar to that of metals. The detailed report on the hydrogen storage properties and mechanism of hydride functionalized CNTs was presented in [186].

Details of the investigation results of the part of the above selected works on modification by other doping are presented in Table 4.

## 4. Conclusions

Many of the computer simulation results of hydrogen adsorption in CNTs have some deviations from the experimental data; however, we cannot rule out that some of the experimental measurement methods are not ideal, and the measurement methods are not perfect. Therefore, at present, some results obtained by computer simulation can at least provide a useful reference for the application research of hydrogen storage of CNTs, which show broad prospects of CNTs for hydrogen adsorption. Although there are still many differences, researchers have basically reached the consensus on some conclusions: (1) The adsorption capacity is positively correlated with specific surface area and micropore volume, the adsorption of hydrogen by mesopore and large pore is weak, which are not conducive to hydrogen storage; (2) The hydrogen adsorption occurs inside and outside the tube, or at the gap of the array of bundles; (3) Size, geometric, and arrangement of CNTs exert influence on hydrogen adsorption; (4) Surface activation or doping plays an important and even decisive role in the adsorption properties; (5) Low temperature and high pressure are favorable for hydrogen adsorption in CNTs; (6) Extent of activation depends not only on activation temperature but also on chemical activator; (7) High doping amount of metals will lead to the clustering of metal nanoparticles. Small size and large dispersion of metal particles are beneficial to hydrogen absorption; (8) The reversible hydrogen adsorption capacity of CNTs at room temperature can be improved by properly increasing *E_ad_* of hydrogen; (9) High irradiation dose will destroy the microporous structure, resulting in a decrease in hydrogen adsorption capacity. At present, there are two recognized hydrogen adsorption mechanisms of metal-doped CNTs: spillover mechanism and the Kubas interaction; however, there are still many inconsistencies between the experimental results and the theoretical simulation results. Therefore, H_2_ adsorption on metal-functionalized CNTs still lacks a consistent theory.

According to current reports, CNTs with high hydrogen adsorption capacity can be obtained in the following ways: (1) Co-doping of metal (hydride, oxide) and hetero-atoms with optimum doping sites and doped configurations. It is worth emphasizing that Pd and Al are dopants that can effectively improve hydrogen adsorption capacity, and Co is a dopant that allows good cycle stability of hydrogen absorption/desorption. In addition, B and N can inhibit metal atom from clustering on the surface of the CNTs; (2) The hydrogen adsorption capacity of CNTs can be further increased by positively charging, mechanical bending, or activating CNTs. We believe that there are two problems in the design of CNTs with high hydrogen adsorption capacity: (1) A detailed study of the optimum number of metal atoms without aggregation on CNT should be performed, at the same time suitable preparation methods for realizing controllable doping site and doped configurations should be devised; (2) The material synthesis, purification, and activation methods have to be optimized; (3) Active sites, molecular configurations, effectively accessible surface area, pore size, surface topology, chemical composition of the surface, applied pressure and temperature, defects and dopant, which are some of the important factors that strongly affect the hydrogen adsorption in CNTs, should be better understood.

## Figures and Tables

**Table 1:** Hydrogen storage properties of carbon nanotubes (CNTs) modified by physical and chemical treatments.

Carbon Material	CNTs Synthetic Method	Research Method	Method/Device for Measuring Hydrogen Amount	Hydrogen Amount	Refs.
SWNTs (triangular array)	-	GCMC	-	Ads: 6 wt%/45 kg/m/77 K/1 MPa	[32]
KOH-activated MWCNT	chemicalvapor deposition (CVD)	experimentally	volumetric method	Ads: 1.2 wt%/12 MPa/298 K	[37]
CNT	CVD	experimentally	Hidden IMI PSI gas storage device	Ads: 1.14 wt%/80 bar/77 K	[63]
Triplet form of (5,0) CNT	-	DFT	-	Ads: 10.4 wt% (physisorption 4.4 wt% + chemisorption 6 wt%)	[64]
MWCNT milled without MgO for 2 h	the catalytic decomposition of acetylene	experimentally	volumetric method	Ads: 0.22 wt%/8–9 MPa/298 K	[78]
MWCNT milled without MgO for 10 h	the catalytic decomposition of acetylene	experimentally	volumetric method	Ads: 0.65 wt%/8–9 MPa/ 298 K	[78]
MWCNT milled with MgO for 1 h (The weight ratio of MWNT and MgO was 1:5)	the catalytic decomposition of acetylene	experimentally	volumetric method	Ads: 0.69 wt%/8–9 MPa/298 K	[78]
SWCNT (square array)	-	MD	-	Ads: 1.8 wt%/14 MPa/298 K	[84]
SWCNT (triangular array)	-	MD	-	Ads: 1.6 wt%/14 MPa/298 K	[84]
SWCNT	-	MD	-	Ads: 1.4 wt%/14 MPa/298 K	[84]
SWCNT	arc discharge	experimentally	volumetric method	Ads: 1.73 wt%/10 MPa/77 K Des: 1.23 wt%/1 atm/77 K Ads: 0.95 wt%/10 MPa/203 K Des: 0.62 wt%/1 atm/203 K Ads: 0.67 wt%/10 MPa/303 K Des: 0.42 wt%/1 atm/303 K	[97]
CNT	thermal chemical vapor deposition (TCVD)	experimentally	Elastic Recoil Detection Analysis	Ads: 0.175 wt% /5 bar/30 °C	[99]
KOH and heat-activated CNT (Mass radio of KOH/carbon = 2, activation temperature 800 °C)	-	experimentally	gravimetric method	Ads: 5.8 wt%/20 bar/77 K	[107]
KOH and heat-activated CNT (Mass radio of KOH/carbon = 4, activation temperature 800 °C)	-	experimentally	gravimetric method	Ads: 7.3 wt%/20 bar/77 K	[107]
MWCNT (150 kGy γ-irradiation in air)	-	experimentally	-	Ads:1.2 wt%/1 atm/100 °C	[108]
microwave-treated MWCNT	CVD	experimentally	-	Ads: 0.35 wt%/298 K	[109]
Microwave and heat treated MWCNTs	CVD	experimentally	-	Ads: 0.4 wt%/298 K	[109]
MWCNT ball milled for 6 h at −180 °C with the milling speed of 300 rpm	catalytic chemical vapor deposition (CCVD)	experimentally	volumetric method	Ads: 1.815 mg/g /100 kPa/77 K	[111]
MWCNT ball milled for 6 h at –180 °C with the milling speed of 700 rpm	CCVD	experimentally	volumetric method	Ads: 2.215 mg/g /100 kPa/77 K	[111]
Ball milled CNT	arc discharge	experimentally	volumetric method	Ads: 0.9 wt%/2.47 MPa/290 K/4000 s	[112]
MWCNT	TCVD	experimentally	volumetric method	Ads: 0.35 wt%/1 atm/298 K	[115]
Bromine activated MWCNT	TCVD	experimentally	volumetric method	Ads: 1.15 wt.%/1 atm/298 K	[115]
H_2_SO_4_ activated MWCNT	TCVD	experimentally	volumetric method	Ads: 0.41 wt%/1 atm/298 K	[115]
HCl activated MWCNT	TCVD	experimentally	volumetric method	Ads: 0.62 wt%/1 atm/298 K	[115]
HNO_3_ activated MWCNT	TCVD	experimentally	volumetric method	Ads: 0.85 wt%/1 atm/298 K	[115]
MWCNT	-	experimentally	volumetric method	Ads: 0.42 wt%/10 MPa/30 °C	[116]
MWCNT activated by Fluorine at 350 °C	-	experimentally	volumetric method	Ads: 1.69 wt%/10 MPa/30 °C	[116]
MWCNT activated by KOH at 900 °C	CCVD	experimentally	volumetric method	Ads: 1.24 wt%/34 bar/298 K	[118]
As purified MWCNT	CCVD	experimentally	volumetric method	Ads: 0.67 wt%/34 bar/298 K	[118]
MWCNT activated by H_2_SO_4_:HNO_3_ = 3:1	CCVD	experimentally	volumetric method	Ads: 0.40 wt%/34 bar/298 K	[118]
SWCNT with 80% purity	CVD	experimentally	-	Ads: 0.4 wt%	[124]
SWCNT with 90% purity	CVD	experimentally	-	Ads: 0.5 wt%	[124]
SWCNT	pyrolysis method	experimentally		Ads: 8 wt%/2 MPa/290 K	[126]

^1^ CVD—chemical vapor deposition, GCMC—Grand canonical Monte Carlo, DFT—density functional theory, MD—molecular dynamics, TCVD—thermal chemical vapor deposition, CCVD—catalytic chemical vapor deposition, CNT—carbon nanotube, SWCNT—single-walled carbon nanotube, MWCNT—multi-walled carbon nanotube.

**Table 2:** Hydrogen storage properties of CNTs modified by metal dopants.

Carbon Material	CNTs Synthetic Method	Doping Method	Research Method	Method/Device for Measuring Hydrogen Amount	Hydrogen Amount	Refs.
100 mg MWCNT-1 mol Pd	TCVD	solution method	experimentally	volumetric method	Abs: 7 wt%/1 atm/298 K	[47]
100 mg MWCNT-1 mol Ni	TCVD	solution method	experimentally	volumetric method	Abs: 0.4 wt%/1 atm/298 K	[47]
100 mg MWCNT-1 mol Fe	TCVD	solution method	experimentally	volumetric method	Abs: 0.75 wt%/1 atm/298 K	[47]
100 mg MWCNT-1 mol Co	TCVD.	solution method	experimentally	volumetric method	Abs: 1.5 wt%/1 atm/298 K	[47]
100 mg MWCNT-1 mol Ca	TCVD	solution method	experimentally	volumetric method	Abs: 1.05 wt%/1 atm/298 K	[47]
MWCNT	TCVD	solution method	experimentally	volumetric method	Abs: 0.3 wt%/1 atm/298 K	[47]
(8,0) SWCNT-8(Al + 4$H_{2}$)	-	-	DFT	-	Abs: 6.15 wt% *E_ad_* = 0.214 eV/H_2_	[102]
MWCNT-3.72 wt% Pt	CVD	chemical reduction	experimentally	volumetric method	Abs: 18 cm^3^/g /100 bar/298 K	[122]
Nitric-activated MWCNT-1 wt% Pd	CVD	reverse micro-emulsion method	experimentally	volumetric method	Abs: 0.91 wt%/50 bar/123 K; Abs: 0.45 wt%/50 bar/223 K; Abs: 0.24 wt% /50 bar/303 K	[130]
Nitric-activated MWCNT-5 wt% Pd	CVD	reverse micro-emulsion method	experimentally	volumetric method	Abs: 1.16 wt%/50 bar/123 K; Abs: 0.81 wt%/50 bar/223 K; Abs: 0.49 wt% /50 bar/303 K	[130]
Nitric-treated MWCNT-10 wt% Pd	CVD	reverse micro-emulsion method	experimentally	volumetric method	Abs: 1.25 wt%/50 bar/123 K; Abs:1.05 wt%/50 bar/223 K; Abs: 0.64 wt% /50 bar/303 K	[130]
Sc-doped capped-SWCNT C_30_(Sc)_6_(H_2_)_24_	-	-	DFT	-	Abs: 7.08 wt% *E_ad_* = 0.93 eV/H_2_	[134]
Chemical-activated MWCNT	catalyzed vapor decomposition	electroless deposition	experimentally	gravimetric method	Abs: 0.35 wt%/6.89 MPa/298 K	[139]
Chemical-activated MWCNT-9.2 wt% Ni	catalyzed vapor decomposition	electroless deposition	experimentally	gravimetric method	Abs: 1.02 wt%/6.89 MPa/298 K	[139]
MWCNT + 1.2 wt% Li	-		experimentally	volumetric method	Abs: 3.9 wt%/106.66 kPa/77 K	[144]
Capped CNT-6(Y + 6$H_{2}$)	-	-	DFT	-	Abs: 7.51wt% *E_ad_* = 0.48eV/H_2_	[145]
SWCNT-4(Y + 6$H_{2}$)	-	-	DFT	-	Abs: 6.1 wt%/300 K *E_ad_* = 0.31eV/H_2_	[146]
Li-doped CNT with the configuration of eight Li dispersed at the hollow sites above the hexagonal carbon rings	-	-	DFT	-	Abs: 13.45 wt% *E_ad_* = 0.17 eV/H_2_	[150]
Nitric-activated MWCNT-11.54 wt% Pd	-	Chemical reduction	experimentally	volumetric method	Abs: 1.1 wt% /1.5 bar/298 K	[151]
Nitric-activated MWCNT-57.7 wt% Pd	-	laser ablation	experimentally	volumetric method	Abs: 6 wt%/1.5 bar/298 K	[151]
Nitric-activated MWCNT + 5 wt% Pd	CVD	polyol methods	experimentally	IMI analyzer	Abs: 6 wt%/50 atm/123 K	[152]
Nitric-activated MWCNT + 5 wt% Pd	CVD	wet impregnation	experimentally	IMI analyzer	Abs: 0.7 wt% /50 atm/123 K	[152]
DWCNT-2 wt% Pd	-	Chemical reduction at 300℃ under H_2_ atmosphere	experimentally	Sieverts method	Abs: 1.85 wt% /1 atm/298 K	[153]
DWCNT-2 wt% Pd	-	Chemical reduction at 400℃ under H_2_ atmosphere	experimentally	Sieverts method	Abs: 2 wt% /1 atm/298 K	[153]
DWCNT-2 wt% Pd	-	Chemical reduction at 500℃ under H_2_ atmosphere	experimentally	Sieverts method	Abs: 1.93 wt% /1 atm/298 K	[153]
DWCNT	-	Chemical reduction	experimentally	AMC Gas Reactor Controller	Abs: 1.7 wt%1 atm/298 K	[155]
DWCNT-1 wt% Pd	-	Chemical reduction	experimentally	AMC Gas Reactor Controller	Abs: 1.85 wt%1 atm/298 K	[155]
DWCNT-2 wt% Pd	-	Chemical reduction	experimentally	AMC Gas Reactor Controller	Abs: 3 wt%1 atm/298 K	[155]
DWCNT-3 wt% Pd	-	Chemical reduction	experimentally	AMC Gas Reactor Controller	Abs: 2 wt%/1 atm/298 K	[155]
MWCNT	TCVD	-	experimentally	volumetric method	Abs: 1.4 wt%	[161]
MWCNT-10.4 wt% Mg	TCVD	-	experimentally	volumetric method	Abs: 1.8 wt%	[161]
Nitric-activated MWCNT-12.3 wt% Ni	-	Chemical reduction	experimentally	volumetric method	Abs:0.6 wt% /1.5 bar/30 °C	[163]
Nitric-activated MWCNT-12.3 wt% Ni	-	laser ablation	experimentally	volumetric method	Abs: 1 wt%/1.5 bar/30 °C	[163]
KOH-activated CNT	CVD	Chemical reduction	experimentally	volumetric method	Abs: 0.44 wt%/100 bar/298 K	[164]
KOH-activated CNT-1.2 wt% Ni	CVD	Chemical reduction	experimentally	volumetric method	Abs: 0.65 wt%/100 bar/298 K	[164]
KOH-activated CNTs-2.2 wt% Ni	CVD	Chemical reduction	experimentally	volumetric method	Abs: 0.74 wt%/100 bar/298 K	[164]
KOH-activated CNTs + 4.1 wt% Ni	CVD	Chemical reduction	experimentally	volumetric method	Abs: 0.48 wt%/100 bar/298 K	[164]
Ti-doped CNTs with the configuration where two carbon atoms of the 6-member rings are substituted by a Ti atom	-	-	DFT and MD	-	Abs: 7.75 wt% (209 g H_2_/L)/650 atm/298 K *E_ad_* = 0.649 eV/H_2_	[165]
Al-(7, 7) SWCNT Al_7_C_70_	-	-	DFT	-	Abs: 28 wt% *E_ad_* = 0.131 eV/H_2_	[168]
AlH_3_-(5, 5) SWCNT $C_{5}{\mathrm{AlH}}_{3}$	-	-	DFT	-	Abs: 8.3 wt% *E_ad_* = 0.1–0.2 eV/H_2_	[169]
Super Diamond CNT with 67.8 Ǻ distance between adjacent centers of CNT junctions	-	-	ab-initio and GCMC	-	Ads: 8.35 wt%(9.8 g/L)/100 bar/300 K	[173]
HNO_3_-activated MWCNT + 10 wt% Pd	-	reflux method	experimentally	Sievert’s apparatus	Abs: 0.125 wt% /65 bar/20 °C	[174]
HNO_3_-activated MWCNT + 10 wt% V	-	-	experimentally	Sievert’s apparatus	Abs: 0.1 wt% /65 bar/20 °C	[174]

^2^ DWCNT—double-walled carbon nanotubes.

**Table 3:** Hydrogen storage properties of CNTs modified by hetero-atoms.

Carbon Material	Carbon Source	Research Method	Method/Device for Measuring Hydrogen Amount	Hydrogen Amount	Refs.
Si MWCNT	methane	experimentally	gravimetric method	Abs: 0.3 wt%/100 bar/298 K	[66]
CNT-1.5 at% N	melamine	experimentally	volumetric method	Abs: 0.17 wt% 19 bar/298 K	[175]
CNT	polyphenylacetylene polymer	experimentally	-	Abs: 0.61 wt%	[176]
CNT-6.4 at% N	polypyrrole	experimentally	-	Abs: 1.2 wt%	[176]
BCNT	1,4- divinylbenzene and diborane	experimentally	-	Abs: 2.03 wt%	[176]
CNT-8.5 at% N	polystyrene and polypyrrole	experimentally	volumetric method	Abs: 2 wt%/100 bar/298 K	[177]
CNT-5.4 at% N	imidazole	experimentally	IMI analyzer	Abs: 0.8 wt%/50 bar/163 K	[178]
SWCNT	-	GCMC	-	Abs: 1.4 wt%/100 bar/298 K	[179]
SWCNT-10 atom% Si	-	GCMC	-	Abs: 2.5 wt%/100 bar/298 K	[179]

^3^ BCNT—boron containing carbon nanotube, Si MWCNT—silicon containing multi-walled carbon nanotube.

**Table 4:** Hydrogen storage properties of CNTs modified by other dopants.

Carbon Material	Research Method	Method/Device for Measuring Hydrogen Amount	Hydrogen Amount	Refs.
BCNT-1Ru- 4H_2_	DFT	-	*E_ad_* = 1.151 eV/H_2_	[182]
(4ND)_10_-NCNT-10(Sc + 5H_2_)	generalized gradient approximation (GGA), DFT and MD	-	Ads: 5.85 wt%/300 K *E_ad_* = 0.166 eV/H_2_	[149]
SWCNT-BH_3_	experimentally	CHN-elemental analysis	Ads: 4.77 wt%/50 °C Des temperature: 90–125 °C	[185]
(5,5) SWCNT-5(LiH + H_2_)	DFT	-	Ads: 1.90 wt% *E_ad_* = 0.20 eV/H_2_	[187]
(5,5) SWCNT-5(LiH + 5H_2_)	DFT	-	Ads: 7.36 wt% *E_ad_* = 0.14 eV/H_2_	[187]
(5,5) SWCNT-10(LiH + H_2_)	DFT	-	Ads: 3.48 wt% *E_ad_* = 0.15 eV/H_2_	[187]
(5,5) SWCNT-10(NiH_2_ + H_2_)	DFT	-	Ads: 0.73 wt% *E_ad_* = 0.19 eV/H_2_	[187]
(5,5) SWCNT-10(NiH_2_ + 5H_2_)	DFT	-	Ads: 2.44 wt% *E_ad_* = 0.06 eV/H_2_	[187]
(5,5) SWCNT-5(NiH_2_ + H_2_)	DFT	-	Ads: 1.27 wt%	[187]
SWCNT-BH_3_	experimentally	CHNS elemental analysis	Ads: 1.5 wt%	[188]
(5,5) SWCNT-5(NH_3_ + 5H_2_)	DFT	-	Ads: 8.18 wt% *E_ad_* = 0.091eV/H_2_ Desorption temperature: 115 K	[189]
(5,5) SWCNT-10(NH_3_ + 5H_2_)	DFT	-	Ads: 13.2 wt% *E_ad_* = 0.092eV/H_2_ Desorption temperature: 115 K	[189]
(10,10) SWCNT-8(TiO_2_ + 7H_2_)	DFT	-	Ads: 6.6 wt%	[190]
(10,10) SWCNT-4(TiO_2_ + 6H_2_)	DFT	-	Ads: 3.64 wt% *E_ad_* = 0.25eV/H_2_ Desorption temperature: 332 K	[191]

^4^ GGA—generalized gradient approximation, NCNT—nitrogen doped carbon nanotube, ND—divacancy.
